# Increasing variability of body mass and health correlates in Swiss conscripts, a possible role of relaxed natural selection?

**DOI:** 10.1093/emph/eoy012

**Published:** 2018-04-28

**Authors:** Kaspar Staub, Maciej Henneberg, Francesco M Galassi, Patrick Eppenberger, Martin Haeusler, Irina Morozova, Frank J Rühli, Nicole Bender

**Affiliations:** 1 Institute of Evolutionary Medicine, University of Zurich, Zurich, Switzerland; 2 Adelaide Medical School, University of Adelaide, Adelaide, Australia

**Keywords:** body mass variability, relaxed natural selection, metabolic parameters, inflammatory parameters, underweight, overweight

## Abstract

**Background and objectives:**

The body mass index (BMI) is an established anthropometric index for the development of obesity-related conditions. However, little is known about the distribution of BMI within a population, especially about this distribution’s temporal change. Here, we analysed changes in the distribution of height, weight and BMI over the past 140 years based on data of Swiss conscripts and tested for correlations between anthropometric data and standard blood parameters.

**Methods:**

Height and weight were measured in 59 504 young Swiss males aged 18–19 years during conscription in 1875–79, 1932–36, 1994 and 2010–12. For 65% of conscripts in 2010–12, results of standard blood analysis were available. We calculated descriptive statistics of the distribution of height, weight and BMI over the four time periods and tested for associations between BMI and metabolic parameters.

**Results:**

Average and median body height, body weight and BMI increased over time. Height did no longer increase between 1994 and 2010–12, while weight and BMI still increased over these two decades. Variability ranges of weight and BMI increased over time, while variation of body height remained constant. Elevated levels of metabolic and inflammatory blood parameters were found at both ends of BMI distribution.

**Conclusions and implications:**

Both overweight and underweight subgroups showed similar changes in inflammation parameters, pointing toward related metabolic deficiencies in both conditions. In addition to environmental influences, our results indicate a potential role of relaxed natural selection on genes affecting metabolism and body composition.

## Teaser

The distribution of height, weight, and body mass index of Swiss conscripts widened over the last 140 years. There are now more underweight and overweight conscripts who show similar increases in inflam-matory and metabolic fault blood parameters. Both facts indicate a possible role of relaxed natural selection on genes affecting metabolism and body composition.

## INTRODUCTION

Prevalences of obesity and its unfavourable health correlates, such as diabetes type II, coronary heart disease and certain types of cancer are still increasing on the global scale [1]. In Switzerland, the mean BMI of children [2, 3] and male conscripts [4, 5] increased throughout the twentieth century, although it seems to have steadied in the last few years. Nevertheless, little is known about the distribution of BMI within a population, especially about this distribution’s change over time. In Swiss conscripts distributions of weight and BMI experienced an increasing right shift during the last hundred years, indicating a disproportional increase in overweight and obese young males [5]. However, the lower end of the BMI distribution is generally less well studied in affluent countries, probably because underweight is not a focus of public attention while corresponding unfavourable health correlates are rare in such countries.

Overweight and obesity are correlated to increased morbidity and mortality in later life, and also to changes of biomarkers such as plasma lipid profiles or blood glucose towards pathological levels [6]. These biomarkers are therefore relevant screening parameters for the prevention of coronary heart disease and type II diabetes. Furthermore, overweight and obesity are correlated to a chronic state of systemic inflammation, which is regarded as causally linked to the unfavourable health outcomes mentioned above [7]. There are indications that not only overweight, but also underweight, in well-nourished populations, is associated to an increased overall morbidity [8] and mortality [9, 10]. Yet, little is known about the prevalence of pathologic changes in blood markers at the lower end of BMI distribution in European affluent populations.

Several causes of the obesity epidemic have been discussed in the literature, such as an imbalance between energy input and output, sleep deprivation, gut microbiome, epigenetics, etc. [11, 12]. Besides environmental factors, genetic causes are considered to be relevant for the obesity epidemics. To date, little attention has been given to the phenomenon of relaxed natural selection in modern human civilizations due to advances in medicine and improved hygiene, leading to an increased survival and a subsequent reduction of differential reproduction of different genotypes [13]. The biological state index (*I*_bs_) is an index of the relaxed opportunity for natural selection in a given population [14–16]. Essentially, this index measures the opportunity for an individual born into a given population to pass on genes to the next generation. It combines life table function *d_x_* (number of deaths at age *x*) with the age-specific fertility rate *s_x_* (average number of live births to a woman up to age *x*): *I*_bs_ = 1 – Σ*d_x_s_x_*. This is an improvement on what James Crow [17] called *P*_s_ (proportion surviving) when constructing his index of the opportunity for natural selection. This index took account only of pre-reproductive mortality (*P*_d_ = 1 −- *P*_s_) while the *I*_bs_ also includes mortality during the reproductive life span, weighted by the relative reproductive performance up to the age at death. The *I*_bs_ measures the opportunity for selection through differential mortality. The additional opportunity for selection through differential fertility is not considered because heritable variance of actual fertility in humans is very low. In 1525 historical American and Polish couples of completed fertility who did not control family size (non-Malthusian), the heritable variance of fertility was less than 0.01 [18]. Birth control has been practiced for over 100 years becoming widespread in the last two generations thus further reducing fertility differentials related to genetic background of parents. Presently, lifetime reproductive success, as measured by the total number of children born to a parent [19, 20] may be differentiated in relation to phenotypes and genotypes, but it cannot capture a change of balance between mutation and selection, which would require comparison to previous generations. Genetic modelling shows a very strong accumulation of random germline mutations since mortality has been reduced in modern times [21]. The reduction of natural selection accelerates at present genetic change to a greater extent than previously thought, with a high mutation rate for somatic as well as germline cells [22, 23]. As a consequence, a substantial and rapid accumulation of mutations may have altered genes affecting energy balance and metabolism over only a few generations, contributing to the observed increase of obesity [23, 24].

For Switzerland, the *I*_bs_ was 0.993 in 2006, one of the highest in the world, therefore the probability of an average individual born into this population to fully participate in the reproduction of the next generation and to pass her/his genes to the next generation was 99.3%. At the same time, the probability of accumulation of random mutations was very high. Of course, due to Malthusian fertility, the probability to pass genes to the next generation may not be used by some individuals or may be reduced by personal choices. Such effects cannot be measured at the population level.

In this study, we follow two aims: first, we want to analyse changes in height, weight and BMI distribution of Swiss conscripts over the last 140 years as the conscripts cover >90% of the local male birth cohorts. Particularly, we want to focus on the lower end and the breadth of BMI distribution. Second, we aim to test for associations between anthropometric variables and blood parameters of volunteering conscripts from recent years. This will highlight the comparison of underweight versus overweight subgroups.

## MATERIALS AND METHODS

The presented data combine *N* = 159 504 young Swiss males aged 18–19 years who had their body height and weight measured during conscription to the Swiss armed forces in 1875–79, 1932–36, 1994 and 2010–12. For approximately 65% of conscripts examined in 2010–12, information on results of standard blood analysis was available. The anthropometric and metabolic data re-analysed here have been previously published and described in detail elsewhere [4, 5, 25, 26]. Also, the process of Swiss conscription and voluntary blood sampling during medical examination has been largely described in these publications. In brief, conscription in Switzerland is compulsory for all Swiss male citizens at the age of 19 years. Earlier or later conscription is possible upon request. Conscription process lasts for 2–3 days and is carried out in one of six centres distributed across Switzerland. In the course of medical examination height and weight measurements are taken of all conscripts, regardless of later assessed fitness for service. Anthropometric measurements are taken using stadiometers and scales (both SECA©), in underwear and without shoes. All examinations are carried out in identical manner in all six centres by trained medical staff following uniform protocols, which remained unchanged over time, and using calibrated instruments. Anthropometric data have been shown to have high coverage and include almost full male birth cohorts for historic data [5, 27] and still over 90% of a given male birth cohort in modern times [4, 26, 28]. The medical causes of absences of the remaining up to 10% conscripts include the entire range of severe diseases and severe physical and psychiatric disabilities.

Since 2004, a voluntary blood exam is offered to the conscripts, including blood cell counts, liver enzymes, haemoglobin, glucose, C-reactive protein (CRP), lipid profile, ferritin, uric acid, and creatinine. Blood samples are taken by medical personnel at conscription centres and transferred to a central laboratory in Allschwil (Viollier AG) to be tested by state-of-the-art equipment and assays, usually within 12 h, by laboratory personnel. Approximately 65% of the conscripts consent to participate in the blood test. In an earlier publication on a similar data set [25], participants and non-participants have been shown to be comparable in age composition and socio-economic background. However, there are differences in a low single-digit percentage range in terms of prevalence of overweight/obesity, physical fitness performance and place of residence.

Approximately 80% of Swiss conscripts have to undergo a physical fitness exam (Test Fitness Rekrutierung, TFR), which assesses strength and endurance through five athletic disciplines (speed, strength of upper and lower extremities, global trunk strength, coordination and endurance) [29–31]. Performance is evaluated for each athletic discipline using a point scale (0–25 points per discipline, 125 points maximum in total). For this study, the total of achieved points, indicating overall performance, was available.

For the present study, we only included conscripts appearing for the first, regular assessment in the recruitment centres, and we excluded conscripts older than 22 years and appearing for conscription with severe delay (<2% of the original data). The data set was checked for implausible height, weight, physical fitness exam results and blood parameter values, but none were found. We calculated BMI (weight [kg]/height [m]2) and age at conscription based on date of birth and date of conscription. BMI was categorized according to the official WHO subgroups for underweight (BMI < 18.5 kg/m^2^), normal weight (BMI 18.5–24.9 kg/m^2^), overweight (BMI 25.0–29.9 kg/m^2^) and obesity (BMI ≥ 30.0 kg/m^2^), including the official subcategories. Assessed blood parameter values were categorized for elevated or reduced values according to standard thresholds. The final data set included *N* = 159 504 conscripts (*N* = 7937 in 1875–79, *N* = 15 707 in 1932–39, *N* = 25 050 in 1994). For *N* = 95 467 (86.2%) of the total *N* = 110 810 conscripts with available height and weight measurements from 2010 to 2012 results of the physical fitness exam were available, and blood analysis results were available for *N* = 71 707 (64.7%) to *N* = 72 207 (65.2%), depending on the parameter. However, the number of conscripts with a recorded result was lower for the fasting glucose (*N* = 57 702 or 52.1%). Assessed blood parameters included cell counts of erythrocytes, thrombocytes, leukocytes, neutrophils, lymphocytes, monocytes, eosinophils and basophils; as well as serum concentrations of CRP, total cholesterol (TCL, sub-fractions were not available), ferritin, haemoglobin, fasting glucose, alanine transaminase (ALT) and creatinine.

The 1994 and 2010–12 anthropometric and metabolic data were fully anonymized by the Swiss Army (Logistikbasis der Armee—Sanität) and handed over to the study authors under contractual agreement. The Swiss Armed Forces are authorized to provide the anonymized data for academic research according to Swiss federal law (Bundesgesetz über die militärischen Informationssysteme MIG, BG 510.91, Art. 2, 9, 24–29). The conscripts signed a detailed informed consent form for the voluntary laboratory test (available from the Swiss Army upon request). Because Swiss conscription is mandatory and the anthropometric measurements used in this study are fully anonymized governmental data, ethical approval was not needed (Swiss data privacy act, SR 235.1; 19.6.1992 and Federal Act on Research involving Human Beings HRA, 810.30; 1.1.2014) [4, 25, 26].

To compare the changes in distribution of height, weight and BMI over the four periods relevant descriptive statistics for continuous and categorized data, and kernel density plots and box plots were produced. To visually assess changes of BMI distribution width over time, for each value the absolute difference (positive or negative) from the median for all time periods was calculated. Absolute differences from the median were log-transformed due to right-skewed distribution for modern data. Correlations between all metabolic and other variables at hand for the 2010–12 data were calculated as Pearson correlation coefficients and Spearman’s rho (as a rank-based measure of association) and displayed as alphabetically ordered correlation heatmap matrices in the Supplementary Appendix using the ‘corrplot’ R package [32]. Associations between BMI and metabolic parameters were furthermore explored using a linear regression model with metabolic parameters as a dependent variable and categorized BMI as an independent variable (20.0–22.4 kg/m^2^ as reference category). Results are presented as coefficient plots. Statistical analysis and figures were performed using Stata (Version 14.2, StataCorp LP, Texas, USA) and R [33].

## RESULTS

**Figure 1.**
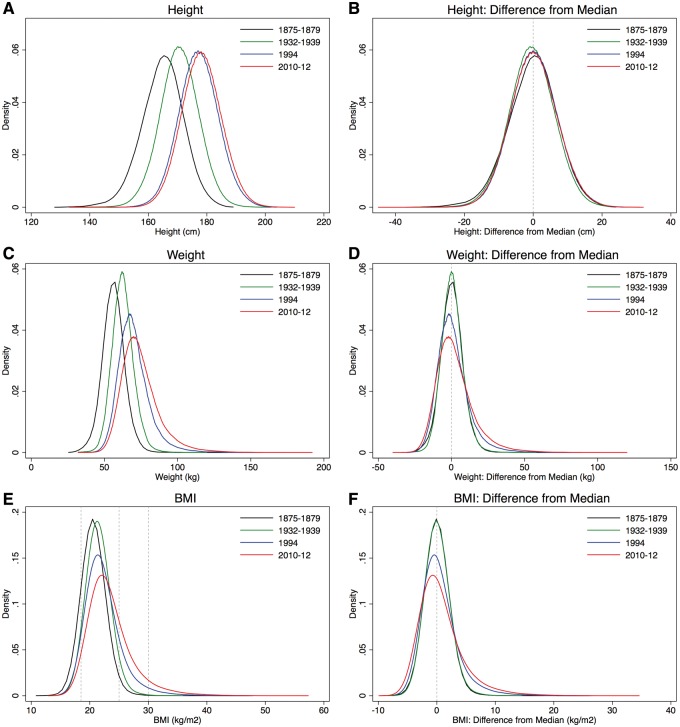
Kernel density plots of height, weight and BMI (left), and distributions of differences from the median (right), per years of conscription

Average and median body height, body weight and BMI increased over time. Young men in Switzerland became taller and heavier (Table 1), but while height did no longer increase between 1994 and 2010–12, the conscripts’ weight and BMI still increased over these two decades. As indicated by the boxplots (Supplementary Appendix Fig. S1) and kernel density plots (Fig. 1C and E) distributions of weight and BMI became progressively wider and right-skewed during the last 80 years. Modal frequencies decreased. This is reflected in the prevalence of underweight, overweight and obese conscripts: There was practically no change in the fraction of conscripts with BMI < 18.5 kg/m^2^ (5% in 1932–39 vs. 5% in 1994 vs. 4% in 2010–12), while fractions of overweight (4% in 1932–39 vs. 12% in 1994 vs. 20% in 2010–12) and obese young males (0% in 1932–39 vs. 3% in 1994 vs. 6% in 2010–12) increased substantially (Table 2). These results are confirmed when considering the WHO-subcategories for underweight and obesity.

**Table 1.** Parameters of distributions of height, weight and BMI of the included conscripts

	Height (cm)						Weight (kg)				BMI (kg/m^2^)					
YoC	A: 1875–79	B: 1932–39	C: 1994	D: 2010–12	% (A) > (D)	% (B) > (D)	1875–79	1932–39	1994	2010–12	A: 1875–79	B: 1932–39	C: 1994	D: 2010–12	% (A) > (D)	% (B) > (D)
*N*	7937	15 707	25 050	110 810			7937	15 707	25 050	110 810	7936	15 707	25 050	110 810		
Mean	164.81	170.59	177.26	178.21	108.1	104.5	56.18	62.52	70.08	74.51	20.63	21.46	22.28	23.44	113.6	109.2
SE mean	0.079	0.051	0.041	0.020			0.084	0.057	0.068	0.039	0.022	0.016	0.019	0.011		
Median	165	171	177	178	107.9	104.1	56	62	69	72	20.56	21.36	21.80	22.74	110.6	106.5
Min	130	136	146	133	102.3	97.8	27.5	32	35	32	11.83	13.50	14.71	12.84	108.5	95.1
Max	187	202	204	210	112.3	104.0	143	120	158	192	46.16	35.83	47.88	57.33	124.2	160.0
P5	153	160	167	168	109.8	105.0	44	51	56	58	17.64	18.50	18.41	18.79	106.5	101.6
P25	160.3	166.5	173	174	108.5	104.5	51.5	58	63	66	19.38	20.16	20.29	20.96	108.1	103.9
P75	169	175	182	183	108.3	104.6	61	67	75	81	21.79	22.64	23.63	25.06	115.0	110.7
P95	176	181	188	189	107.4	104.4	68	75	89	99	23.78	24.74	27.78	30.59	128.6	123.6
Skew	−0.34	−0.09	0.05	0.08			0.37	0.43	1.28	1.36	0.93	0.55	1.53	1.55		
Kurt	3.65	3.64	3.17	3.18			6.50	4.65	6.78	6.74	10.35	4.64	7.77	7.33		
SD	7.00	6.44	6.50	6.55			7.47	7.19	10.70	13.08	1.97	1.94	3.05	3.78		
Var	48.98	41.43	42.23	42.87			55.78	51.71	114.47	171.08	3.88	3.78	9.32	14.29		

YoC, year of conscription.

**Table 2.** Distributions of conscripts into BMI categories, in absolute numbers (above) and percentages (below)

BMI categories according to WHO—absolute frequencies (*N*)												Ratio	
YoC	<16.0	16.0–16.9	17.0–18.4	18.5–19.9	20.0–22.4	22.5–24.9	25.0–27.4	27.5–29.9	30.0–34.9	35.0–39.9	>=40.0	<18.5/18.5–24.9	
1875–79	50	121	795	2045	3775	1006	114	18	8	3	1	0.14	
1932–39	15	68	700	2706	7941	3667	514	78	16	2		0.05	
1994	34	162	1168	3763	10 126	6209	2211	753	498	103	23	0.07	
2010–12	95	466	3522	11 413	36 277	30 843	15 241	6424	4798	1295	436	0.05	
Ratio 2010–12/1875–79	1.9	3.9	4.4	5.6	9.6	30.7	133.7	356.9	599.8	431.7	436.0		
BMI categories according to WHO—absolute frequencies (percent, %)												Percent (%) of <25.0	
YoC	<16.0	16.0–16.9	17.0–18.4	18.5–19.9	20.0–22.4	22.5–24.9	25.0–27.4	27.5–29.9	30.0–34.9	35.0–39.9	>=40.0	<18.5	18.5–24.9
1875–79	0.6	1.5	10.0	25.8	47.6	12.7	1.4	0.2	0.1	0.0	0.0	12.4	87.6
1932–39	0.1	0.4	4.5	17.2	50.6	23.3	3.3	0.5	0.1	0.0	0.0	5.2	94.8
1994	0.1	0.6	4.7	15.0	40.4	24.8	8.8	3.0	2.0	0.4	0.1	6.4	93.6
2010–12	0.1	0.4	3.2	10.3	32.7	27.8	13.8	5.8	4.3	1.2	0.4	4.9	95.1
Ratio 2010–12/1875–79	0.1	0.3	0.3	0.4	0.7	2.2	9.6	25.6	43.0	30.9	31.2		

**Figure 2.**
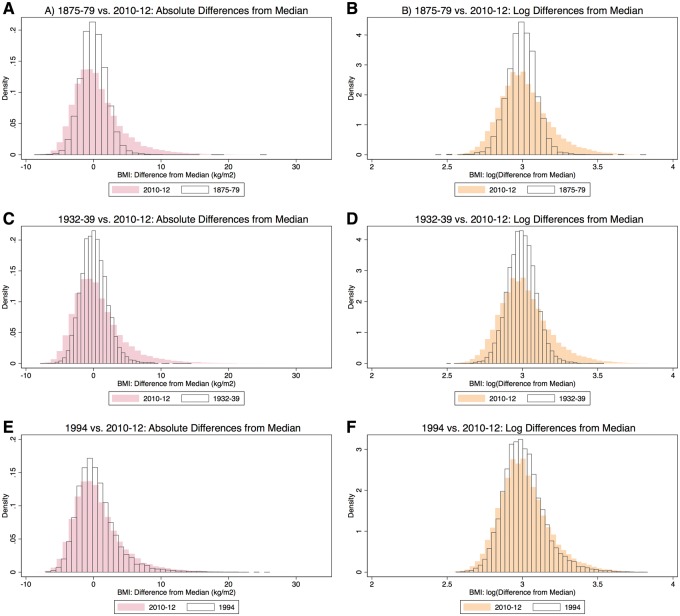
Comparisons of distributions of absolute differences from the median of BMI (left), and log-transformed differences (right). Modern distributions (2010–12) are in colour. In the top row, 1875–79 is compared to modern data, in the middle row, 1932–39 is compared to modern data and in the bottom row, 1994 is compared to modern data

The distributions of the differences from median weight and BMI for each of the time periods and the underlying log-transformed distributions of the differences from median weight and BMI of 2010–12 are shown in Fig. 1. The earlier time periods show that the BMI distribution became broader towards both ends (Fig. 2). This indicates a greater deviation from the median both up and down in more recent times.

**Figure 3.**
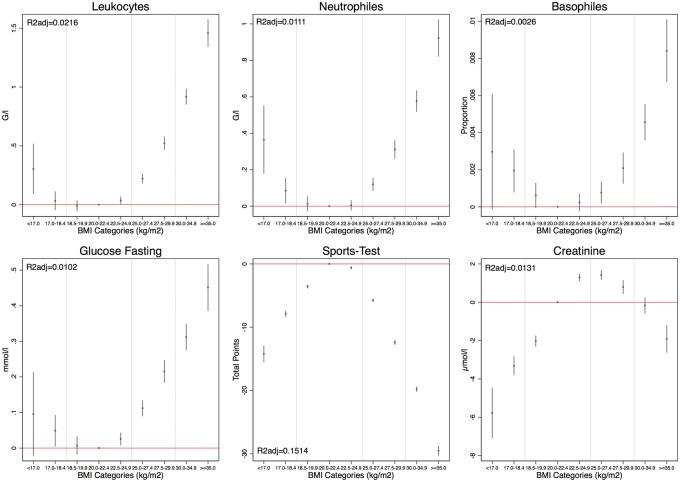
Coefficient plots of linear regression model with metabolic parameters as a dependent variable and categorized BMI as an independent variable (20.0–22.4 kg/m^2^ as reference category)

The overall associations between anthropometric values, sport test and blood analysis parameters in 2010–12 are highlighted in heatmap matrices in Supplementary Appendix Fig. S2 (Pearson’s correlation coefficients) and Fig. 3 (Spearman’s *rho*). The linear regressions showed that overweight and obesity were associated with significantly elevated levels of CRP, TCL, thrombocytes, leukocytes, neutrophils, lymphocytes, monocytes, eosinophils, basophils, erythrocytes, ferritin, haemoglobin, fasting glucose and ALT (Supplementary Appendix Fig. S4). The share of overall variation in the blood parameters explained by BMI (as indicated by adjusted *R*^2^ from the linear regressions) ranged from <1% in the case of thrombocytes, eosinophils, and basophils to >5% in the case of TCL, ferritin, ALT and sports test.

The same pattern is also reflected in the fraction of conscripts with metabolic parameters above the standard thresholds (Supplementary Appendix Tables S1 and S2). Values steadily and significantly increased already for conscripts with BMI 22.5–24.9 kg/m^2^, and continued to do so even more explicitly among overweight and obese conscripts. Physical fitness exam performance significantly decreased among overweight and obese conscripts. Creatinine serum levels, did, however, not change in conscripts with BMI 25.0–34.9 kg/m^2^ compared to those with BMI 20.0–24.9 kg/m^2^.

At the lower end of BMI subgroup distributions, inflammation-related indicators such as CRP, the number of white blood cells, neutrophils and basophils were significantly increased in very low body mass individuals (BMI < 18.5 kg/m^2^). These changes were similar to those in overweight individuals, resulting in U-shaped distributions (Fig. 3). Creatinine values were significantly decreased in underweight conscripts similar to overweight individuals. Furthermore, underweight as well as overweight conscripts presented weaker performance in the physical fitness exam than normal weight individuals. Repeating the regression analyses for conscripts with physical fitness exam passing scores only (thus excluding conscripts with results <35 points, who potentially had a health condition), the results did not change (numbers not shown here).

To estimate the magnitude of associations found we analysed the prevalence of conscripts with blood parameters above the standard clinical thresholds for obese (BMI>=30.0 kg/m^2^) and underweight (BMI < 18.5 kg/m^2^) conscripts. Among underweight conscripts, between 16.3% (fasting glucose) and 0.4% (erythrocytes) did have abnormal levels for all analysed parameters. Among obese conscripts, between 29.1% (ALT) and 1.2% (erythrocytes) showed abnormal levels in all analysed parameters. The largest fractions (>10%) of elevated levels among obese conscripts were observed for CRP (15.7%), TCL (23.9%), leukocytes (14.2%), fasting glucose (23.5%) and ALT (29.1%).

## DISCUSSION

In this study elevated levels of evaluated metabolic and inflammatory blood parameters were mostly found among overweight and obese conscripts. However, the rate of elevated levels of these blood parameters, inflammatory parameters in particular, progressed toward the lower end of BMI distribution as well, thus resulting in a U-shaped distribution. These findings may point toward physiological abnormalities in some individuals with a body mass substantially deviating from the most common range of values in either direction. Physiological shortcomings of many individuals with abnormally low and abnormally high body mass could also explain their weaker performance in the physical fitness exam. A sub-analysis excluding individuals with insufficient physical fitness, and therefore potentially diseased individuals, supports this assumption. However, in the present study we cannot rule out pre-existing diseases in the analysed conscripts, and we could not control for potentially confounding factors such as smoking [34].

As participation rate for the blood test was 65% it is also relevant to assess the representativeness of the tested sample. As mentioned in the Methods section, overall the tested and non-tested samples were similar in terms of age and socio-economic status. However, non-participants were slightly less overweight, more obese and had slightly lower sports test results than the participants. This could indicate that less healthy conscripts participated less in the blood test, rendering the tested sample slightly healthier than the average. An alternative explanation could be that there were more underweight conscripts among the tested sample, lowering the mean BMI of this group. However, the greatest differences between participants and non-participants were found in their regional affiliations: Non-participants were more likely to be residents of western and northern Switzerland and less likely to be from eastern Switzerland, whereas the percentages of participants from the other regions were comparable to those of non-participants. These differences might be explained by a culturally influenced willingness to participate in blood testing rather than health reasons.

Among underweight conscripts, 83.7–99.6% had normal levels for all analysed parameters. In a population-based study in China 16.4% of underweight individuals were metabolically abnormal [8], which is in accordance with our results. Among obese Swiss conscripts 70–99% had normal blood parameter levels, indicating a large fraction of so-called metabolically healthy obese young men. The term ‘healthy obese’ is disputed in the literature, and some authors suspect that the absence of pathologic indicators could just indicate a stage before the onset of disease [35]. This is of special relevance in a representative population sample of very young individuals such as conscripts. Additionally, we found a relatively low share of total variation in the blood parameters explained by BMI (adjusted *R*^2^ <3% in 12 out of 16 variables). Previous publications on TCL or ALT among Swiss conscripts from earlier recruitment years have yielded similar *R*^2^ values [25, 36, 37]. This low share of variation explained by BMI can have several reasons: (i) considerably large random measurement errors in some blood parameters (ca. 30–40% errors for cholesterol or glucose as shown by Whitlock *et al.* [38]). (ii) Short-term individual variation in some of the parameters, which cannot be controlled for with our cross-sectional study design. (iii) Non-symmetrical distributions. (iv) Large sample size with the majority of the conscripts in the normal weight area, while the focus of this study rather was on both ends of the BMI distribution. (v) Other independent variables not available in our data set explaining additional variation in blood parameters, e.g. altitude of the place of residence for haemoglobin. An association between physical fitness, low abdominal adiposity and low inflammation state was described independently of BMI in a large population sample, stressing the relevance of physical activity [39]. An additional effect potentially explaining parts of the observed pattern could be a difference in genetic disease predisposition between diseased and metabolically healthy obese individuals.

This study shows that the width of the distribution and thus variation of BMI in Swiss conscripts increased substantially in the recent few decades, not only toward the higher end, but also toward the lower end. Budnik and Henneberg [24] have found an increase in both overweight and underweight people among Polish conscripts while there was no increase in caloric consumption in Poland during the studied period. Apart from strong environmental and social changes in the last decades, it seems that recent changes in the human gene pool may be contributing to the increasing prevalence of obesity observed worldwide [40]. The decrease in premature mortality during the same time period has led to a relaxation of natural selection, which in turn might have led to an accumulation of harmful mutations [16, 41–43]. In addition the relaxation of natural selection seems to accelerate genetic change to a greater extent than previously thought [22, 44]. Although many *de novo* polymorphisms may be neutral or nearly neutral, their most probable effect is detrimental [45]. Natural selection normally eliminates detrimental characters. Yet, especially in the light of relaxed natural selection, mildly detrimental mutations stand a lesser chance of elimination.

One hypothesis on the evolution of human fatness is based on relaxed natural selection. This is the so-called predation release hypothesis [46]. According to this hypothesis, technical and social evolution allowed hominins around 1.8–2 million years ago to defend themselves effectively against large predators. From this time onward, overweight would not be detrimental to predator escape and therefore not selected against any longer. Furthermore, genetic variants restricting body fatness would not be positively selected either, and fatness would increase due to random mutations and genetic drift [46]. The genetic mechanisms described in this article do not contradict the predation release hypothesis, but rather propose an explanation for the additional increase in overweight and obesity over the last centuries.

It can even be hypothesized that mutations causing metabolic deficiencies stimulating the build-up of fat in the human body will further accumulate in the future. More than 600 genes associated to adiposity were identified so far [47], and it is likely that this list will be extended. However, since mutations occur randomly, they should accordingly as often contribute to increased adiposity as to decreased body mass. It is therefore possible that some genetically based metabolic deficiencies accumulating in the human gene pool decrease adiposity, as much as others are increasing it. However, while a human being’s body mass may almost indefinitely increase, the minimum mass of body tissue required to maintain life is obviously limited. This unequal chance of manifestation might be part of the explanation of the today’s known right-skewness of body mass distribution. While the BMI distribution has widened toward both extremes, most individuals whose BMI lies below the median have a BMI of over 18.5 kg/m^2^. Therefore, it is not the number of underweight people that has increased over time, but rather distribution breadth. While the absolute BMI median significantly shifted to the right, the expanded left distribution mostly includes individuals with a BMI between 18.5 and 25 kg/m^2^.

As undernutrition does virtually no longer exist at a population level in Switzerland, a measurable increase in individuals with BMI below 18.5 kg/m^2^ is not expected. On the other hand, modern social tendencies to favour slim and trained body images could contribute to an increase of the fraction of individuals with BMI in the lower normal range. Similarly, energy imbalance has truly led to increased average body mass and an increasing prevalence of obese individuals, obscuring the relative increase in the number of people with very low body mass. Moreover, there is an understudied phenomenon called constitutional thinness, which describes individuals with a BMI below 18.5 kg/m^2^, but which are otherwise healthy. In particular, they do not show the signs of anorexia nervosa like fear of gaining weight, amenorrhea in women, hormonal imbalances, etc. The prevalence of this condition in Western societies is unknown [48]. At the moment, there is no evidence for a genetic basis of constitutional thinness, but if such a genetic basis exists, this fraction of the population might contribute to the maintenance of very lean people in the population. To test the importance of genetic factors in the revealed trends, further studies of obesity and anti-obesity-related genes as well as epigenetic data and environmental influences should be performed [40, 47, 49].

Further determinants of the large increase in the prevalence of overweight individuals and the smaller increase of people at the lower end of BMI distribution are cultural and environmental factors. To date it is unclear to what extent evolutionary, genetic and environmental factors each contribute to the discussed changes in BMI distributions, so that at the moment all potential explanations have to be considered as equally probable. Foods spreading in modern societies like soy [50] or fructose, and the relevance of physical activity [51, 52], are discussed to contribute to the obesity epidemic beyond caloric intake. Besides energy intake and expenditure, an increasing number of demographic and environmental factors potentially contributing to the modern obesity epidemic are discussed in the literature. Among these factors are infections, increasing maternal age, greater fertility among people with higher adiposity, assortative mating, deprivation of sleep, endocrine disruptors, pharmaceutical iatrogenesis, reduction in variability of ambient temperatures, and intrauterine and intergenerational effects [53].

Other important determinants which are indirectly dependent on environmental influences are epigenetic factors and intestinal microbiome composition. Epigenetic changes causing alterations in gene expression can be inherited or acquired during early lifetime (*in utero* and in infancy) or transformed later by surrounding conditions like diet, stress etc. [54, 55]. The microbiome plays a significant role in nutrient metabolism and spending of energy obtained from food; it can be strongly affected by the individual’s lifestyle, especially by diet and medications [56–58].

To explain increasing numbers of underweight people, cultural trends towards low BMIs and pathological conditions like *anorexia nervosa* are discussed, not only in young women, but also in young men in affluent countries [59]. Non-genetic factors other than social desire for a slim body might additionally contribute to a low BMI. One well-known factor is smoking, but other factors were described as well, especially in the elderly general population, such as depression, dementia, poor oral intake, immobility, chewing and swallowing disorders, as well as dysphagia [60]. Interestingly, some of these factors might also be associated with chronic systemic inflammation.

On the other hand, the increase in BMI variation might reflect phenotypic plasticity as a result of adaptation to changing living conditions. If this is the case, deviation from the existing norm toward both ends might depend on a combination of specific genotypes, types of metabolism, environmental factors and gene–environmental interactions. One example of such a gene–environment interaction is the interaction between the obesity variants of FTO and physical activity [61]. In an ancestral environment requiring a high level of physical activity, a mutation variant increasing the risk of obesity may have been adaptive in times of low food supply. Nowadays, with a decrease in physical activity, the same variant will, therefore, lead to a lower risk for obesity. People carrying the ancestral variant, however, will still tend to have a higher BMI, but they could reduce their obesity risk through additional physical activity [61]. Future studies on BMI distribution should therefore include environmental, genetic and epigenetic data as well as their interactions to disentangle different causative factors and potential biases.

## CONCLUSION

Since the decrease of body mass is physiologically limited by the body’s requirements to sustain life, more abnormalities toward an increased body mass can be observed, producing a right-skewed BMI distribution. Cultural and environmental influences intensify this effect. With a relaxed opportunity for natural selection, an increasing number of individuals within the same environment might produce a body mass that either is too low or too high due to sub-optimal physiological regulation of the energy balance and nutrient metabolism. Our finding of increased inflammation parameters in underweight individuals at a level comparable to overweight individuals needs further exploration to assess clinical relevance. Epidemiological studies should therefore also consider the lower end of BMI distribution in order to monitor the development not only of overweight, but also of underweight.

## Supplementary Material

Supplementary Data

## References

[1] WHO. Obesity and overweight. Fact sheet Geneva. WHO, 2017 http://www.who.int/mediacentre/factsheets/fs311/en/ (14 May 2018, date last accessed).

[2] Aeberli I, Ammann RS, Knabenhans M et al Decrease in the prevalence of paediatric adiposity in Switzerland from 2002 to 2007. Public Health Nutr 2010; 13:806–11. 19772692 10.1017/S1368980009991558

[3] Aeberli I, Henschen I, Molinari L et al Stabilization of the prevalence of childhood obesity in Switzerland. Swiss Med Wkly 2010; 140:w13046. 20349364 10.4414/smw.2010.13046

[4] Panczak R, Zwahlen M, Woitek U et al Socioeconomic, temporal and regional variation in body mass index among 188,537 Swiss male conscripts born between 1986 and 1992. PLoS One 2014; 9:e96721. 24819730 10.1371/journal.pone.0096721 PMC4018351

[5] Staub K, Bender N, Floris J et al From undernutrition to overnutrition: the evolution of overweight and obesity among young men in Switzerland since the 19th century. Obes Facts 2016; 9:259–72. 27544200 10.1159/000446966 PMC5644905

[6] Alberti KG, Eckel RH, Grundy SM et al Harmonizing the metabolic syndrome: a joint interim statement of the International Diabetes Federation Task Force on Epidemiology and Prevention; National Heart, Lung, and Blood Institute; American Heart Association; World Heart Federation; International Atherosclerosis Society; and International Association for the Study of Obesity. Circulation 2009; 120:1640–5. 19805654 10.1161/CIRCULATIONAHA.109.192644

[7] Deng T, Lyon CJ, Bergin S et al Obesity, inflammation, and cancer. Annu Rev Pathol 2016; 11:421–49. 27193454 10.1146/annurev-pathol-012615-044359

[8] Gao B, Zhang L, Zhao M. Underweight but metabolically abnormal phenotype: metabolic features and its association with cardiovascular disease. Eur J Intern Med 2016; 29:46–51. 26703431 10.1016/j.ejim.2015.11.020

[9] Calle EE, Thun MJ, Petrelli JM et al Body-mass index and mortality in a prospective cohort of U.S. adults. N Engl J Med 1999; 341:1097–105. 10511607 10.1056/NEJM199910073411501

[10] Berrington de Gonzalez A, Hartge P, Cerhan JR et al Body-mass index and mortality among 1.46 million white adults. N Engl J Med 2010; 363:2211–9. 21121834 10.1056/NEJMoa1000367 PMC3066051

[11] Voss JD. On food supply and obesity, missing the point is easy. Int J Obes (2005) 2017; 41:1169–70. 10.1038/ijo.2017.101 28442779

[12] Ross SE, Flynn JI, Pate RR. What is really causing the obesity epidemic? A review of reviews in children and adults. J Sports Sci 2016; 34:1148–53. 26400631 10.1080/02640414.2015.1093650

[13] Rühli F, Henneberg M. Biological future of humankind – ongoing evolution and the impact of recognition of human biological variation In: Tibayrenc M, Ayala FJ (eds.). On Human Nature Biology, Psychology, Ethics, Politics, and Religion. London: Elsevier, 2016, 263–75.

[14] Henneberg M, Piontek J. Biological state index of human groups. Przeglad Anthropol 1975; XLI:191–201.

[15] Henneberg M. Reproductive possibilities and estimations of the biological dynamics of earlier human populations. J Hum Evol 1976; 5:41–8.

[16] Saniotis A, Henneberg M. Medicine could be constructing human bodies in the future. Med Hypotheses 2011; 77:560–4. 21783327 10.1016/j.mehy.2011.06.031

[17] Crow JF. Some possibilities for measuring selection intensities in man. Hum Biol 1958; 30:1–13. [published Online First: 1958/02/01] 13513111

[18] Henneberg M. Natural selection through differential fertility in human populations: quantitative evaluation of selection intensity. Przeglad Antropol 1980; 46:21–60.

[19] Beauchamp JP. Genetic evidence for natural selection in humans in the contemporary United States. Proc Natl Acad Sci USA 2016; 113:7774–9. [published Online First: 2016/07/13] 27402742 10.1073/pnas.1600398113 PMC4948342

[20] Sanjak JS, Sidorenko J, Robinson MR et al Evidence of directional and stabilizing selection in contemporary humans. Proc Natl Acad Sci USA 2018; 115:151–6. [published Online First: 2017/12/20] 29255044 10.1073/pnas.1707227114 PMC5776788

[21] Sarraf M, Fernandes H., Woodley of Menie M. Mutation accumulation is still potentially problematic, despite declining paternal age: a comment on Arslan et al. Proceedings B 2017; 285:20172511. 10.1098/rspb.2017.2511 PMC5832703 29467263

[22] Henn BM, Botigue LR, Bustamante CD et al Estimating the mutation load in human genomes. Nat Rev Genet 2015; 16:333–43. 25963372 10.1038/nrg3931 PMC4959039

[23] Lynch M. Mutation and human exceptionalism: our future genetic load. Genetics 2016; 202:869–75. 26953265 10.1534/genetics.115.180471 PMC4788123

[24] Budnik A, Henneberg M. Worldwide increase of obesity is related to the reduced opportunity for natural selection. PLoS One 2017; 12:e0170098. 28107497 10.1371/journal.pone.0170098 PMC5249151

[25] Bruggisser M, Burki D, Haeusler M et al Multivariable analysis of total cholesterol levels in male Swiss Armed Forces conscripts 2006-2012 (N = 174,872). BMC Cardiovasc Disord 2016; 16:43. 26888218 10.1186/s12872-016-0218-2 PMC4756510

[26] Panczak R, Held L, Moser A et al Finding big shots: small-area mapping and spatial modelling of obesity among Swiss male conscripts. BMC Obes 2016; 3:10. 26918194 10.1186/s40608-016-0092-6 PMC4758017

[27] Staub K, Ruhli FJ, Woitek U et al BMI distribution/social stratification in Swiss conscripts from 1875 to present. Eur J Clin Nutr 2010; 64:335–40. 20160753 10.1038/ejcn.2010.7

[28] Panczak R, Moser A, Held L et al A tall order: small area mapping and modelling of adult height among Swiss male conscripts. Econ Hum Biol 2017; 26:61–9. 28284175 10.1016/j.ehb.2017.01.005

[29] Staub K, Wyss T, Lehmann S et al Health status of young men in Switzerland: monitoring results from conscription. Praxis 2015; 104:1203–10. 26953370 10.1024/1661-8157/a002166

[30] Wyss T, Beuchat C, Zehr S et al Physical performance in young men at Swiss Army recruitment 1982 to 2005. Schweiz Zeit Sport 2009; 57:75–7.

[31] Wyss T, Roos L, Beuchat C. Technische Weisungen zum Fitnesstest der Armee FTA für die Rekrutierung. Magglingen: Bundesamt für Sport BASPO, 2017.

[32] Wei T, Simko V. R package “corrplot”: Visualization of a correlation matrix (version 0.84), 2017 https://github.com/taiyun/corrplot (14 May 2018, date last accessed).

[33] R Development Core Team 2018 R: A Language and Environment for Statistical Computing. Vienna, Austria: R Foundation for Statistical Computing.

[34] Stokes A, Preston SH. Smoking and reverse causation create an obesity paradox in cardiovascular disease. Obesity 2015; 23:2485–90. 26421898 10.1002/oby.21239 PMC4701612

[35] Bluher M. Are metabolically healthy obese individuals really healthy? Eur J Endocrinol 2014; 171:R209–19. 25012199 10.1530/EJE-14-0540

[36] Grantham JP, Staub K, Ruhli FJ et al Modern diet and metabolic variance–a recipe for disaster? Nutr J 2014; 13:15. 24502225 10.1186/1475-2891-13-15 PMC3923254

[37] Henneberg M, Ruehli FJ, Gruber P et al Alanine transaminase individual variation is a better marker than socio-cultural factors for body mass increase in healthy males. Food Nutr Sci 2011; 2.

[38] Whitlock G, Clark T, Vander Hoorn S et al Random errors in the measurement of 10 cardiovascular risk factors. Eur J Epidemiol 2001; 17:907–9. [published Online First: 2002/08/22] 12188008 10.1023/a:1016228410194

[39] Wedell-Neergaard AS, Eriksen L, Gronbaek M et al Low fitness is associated with abdominal adiposity and low-grade inflammation independent of BMI. PLoS One 2018; 13:e0190645. [published Online First: 2018/01/18] 29342196 10.1371/journal.pone.0190645 PMC5771585

[40] Albuquerque D, Stice E, Rodriguez-Lopez R et al Current review of genetics of human obesity: from molecular mechanisms to an evolutionary perspective. Mol Genet Genomics 2015; 290:1191–221. 25749980 10.1007/s00438-015-1015-9

[41] Stephan CN, Henneberg M. Medicine may be reducing the human capacity to survive. Med Hypotheses 2001; 57:633–7. 11735325 10.1054/mehy.2001.1431

[42] You WP, Henneberg M. Type 1 diabetes prevalence increasing globally and regionally: the role of natural selection and life expectancy at birth. BMJ Open Diabetes Res Care 2016; 4:e000161. 10.1136/bmjdrc-2015-000161 PMC4780042 26977306

[43] Wenpeng Y, Henneberg M. Cancer incidence increasing globally: the role of relaxed natural selection. Evol Appl 2017; 11:140–152. 29387151 10.1111/eva.12523 PMC5775494

[44] Conrad DF, Keebler JE, DePristo MA et al Variation in genome-wide mutation rates within and between human families. Nat Genet 2011; 43:712–4. 21666693 10.1038/ng.862 PMC3322360

[45] Brace CL. The probable mutation effect. Am Nat 1964; 98:453–5.

[46] Speakman JR. A nonadaptive scenario explaining the genetic predisposition to obesity: the “predation release” hypothesis. Cell Metab 2007; 6:5–12. [published Online First: 2007/07/10] 17618852 10.1016/j.cmet.2007.06.004

[47] Perusse L, Rankinen T, Zuberi A et al The human obesity gene map: the 2004 update. Obes Res 2005; 13:381–490. 15833932 10.1038/oby.2005.50

[48] Estour B, Galusca B, Germain N. Constitutional thinness and anorexia nervosa: a possible misdiagnosis? Front Endocrinol 2014; 5:175. [published Online First: 2014/11/05] 10.3389/fendo.2014.00175 PMC4202249 25368605

[49] Suh JM, Zeve D, McKay R et al Adipose is a conserved dosage-sensitive antiobesity gene. Cell Metab 2007; 6:195–207. 17767906 10.1016/j.cmet.2007.08.001 PMC2587167

[50] Roccisano D, Henneberg M. Soy consumption and obesity. FNS 2012; 03:260–6.

[51] Pereira RM, Botezelli JD, da Cruz Rodrigues KC et al Fructose consumption in the development of obesity and the effects of different protocols of physical exercise on the hepatic metabolism. Nutrients 2017; 9:405. 10.3390/nu9040405 PMC5409744 28425939

[52] Stanhope KL. Sugar consumption, metabolic disease and obesity: the state of the controversy. Crit Rev Clin Lab Sci 2016; 53:52–67. 26376619 10.3109/10408363.2015.1084990 PMC4822166

[53] McAllister EJ, Dhurandhar NV, Keith SW et al Ten putative contributors to the obesity epidemic. Crit Rev Food Sci Nutr 2009; 49:868–913. [published Online First: 2009/12/05] 19960394 10.1080/10408390903372599 PMC2932668

[54] Martinez JA, Milagro FI, Claycombe KJ et al Epigenetics in adipose tissue, obesity, weight loss, and diabetes. Adv Nutr 2014; 5:71–81. [published Online First: 2014/01/16] 24425725 10.3945/an.113.004705 PMC3884103

[55] Dalgaard K, Landgraf K, Heyne S et al Trim28 haploinsufficiency triggers bi-stable epigenetic obesity. Cell 2016; 164:353–64.[published Online First: 2016/01/30] 26824653 10.1016/j.cell.2015.12.025 PMC4735019

[56] John GK, Mullin GE. The gut microbiome and obesity. Curr Oncol Rep 2016; 18:45.[published Online First: 2016/06/04] 27255389 10.1007/s11912-016-0528-7

[57] Parekh PJ, Balart LA, Johnson DA. The influence of the gut microbiome on obesity, metabolic syndrome and gastrointestinal disease. Clin Transl Gastroenterol 2015; 6:e91.[published Online First: 2015/06/19] 26087059 10.1038/ctg.2015.16 PMC4816244

[58] Krajmalnik-Brown R, Ilhan ZE, Kang DW et al Effects of gut microbes on nutrient absorption and energy regulation. Nutr Clin Pract 2012; 27:201–14.[published Online First: 2012/03/01] 22367888 10.1177/0884533611436116 PMC3601187

[59] Zhang C. What can we learn from the history of male anorexia nervosa? J Eat Disord 2014; 2:138. 25671131 10.1186/s40337-014-0036-9 PMC4323236

[60] Tamura BK, Bell CL, Masaki KH et al Factors associated with weight loss, low BMI, and malnutrition among nursing home patients: a systematic review of the literature. J Am Med Dir Assoc 2013; 14:649–55.[published Online First: 2013/05/04] 23639716 10.1016/j.jamda.2013.02.022

[61] Rampersaud E, Mitchell BD, Pollin TI et al Physical activity and the association of common FTO gene variants with body mass index and obesity. Arch Intern Med 2008; 168:1791–7.[published Online First: 2008/09/10] 18779467 10.1001/archinte.168.16.1791 PMC3635949

