# Supplementary material for: Increasing variability of body mass and health correlates in Swiss conscripts, a possible role of relaxed natural selection?
Source: Evol Med Public Health. 2018 Apr 28;2018(1):116–26. doi: 10.1093/emph/eoy012 (PMC6007356; doi:10.1093/emph/eoy012)
Supplement: Supplementary Data [file eoy012_supp.zip › Appendix Tables.docx]

Appendix Table 1: Blood cell counts per BMI category. For each cell type the numbers and percentages per BMI category are given, above and below the threshold

|  |  | Thrombocytes | | | Leukocytes | | | Neutrophils | | | Lymphocytes | | | Monocytes | | | Eosinophils | | | Erythrocytes | | | Basophils | | |
| --- | --- | --- | --- | --- | --- | --- | --- | --- | --- | --- | --- | --- | --- | --- | --- | --- | --- | --- | --- | --- | --- | --- | --- | --- | --- |
| BMI (kg/m2) |  | <360G /l | >=360G /l | Total | <10G /l | >=10G /l | Total | <=8.0G /l | >8.0G/l | Total | <=3.5G /l | >3.5G /l | Total | <0.8G /l | >=0.8G /l | Total | <0.47G /l | >=0.47G /l | Total | 0 | >=6.1T /l | Total | <8% | >=8% | Total |
| <17.0 | N | 257 | 4 | 261 | 237 | 24 | 261 | 249 | 11 | 260 | 254 | 6 | 260 | 252 | 8 | 260 | 252 | 8 | 260 | 258 | 3 | 261 | 235 | 25 | 260 |
|  | % | 98.5 | 1.5 | 100.0 | 90.8 | 9.2 | 100.0 | 95.8 | 4.2 | 100.0 | 97.7 | 2.3 | 100.0 | 96.9 | 3.1 | 100.0 | 96.9 | 3.1 | 100.0 | 98.9 | 1.2 | 100.0 | 90.4 | 9.6 | 100.0 |
| 17.0-18.4 | N | 1938 | 54 | 1992 | 1851 | 141 | 1992 | 1926 | 62 | 1988 | 1966 | 22 | 1988 | 1896 | 92 | 1988 | 1943 | 45 | 1988 | 1985 | 7 | 1992 | 1807 | 181 | 1988 |
|  | % | 97.3 | 2.7 | 100.0 | 92.9 | 7.1 | 100.0 | 96.9 | 3.1 | 100.0 | 98.9 | 1.1 | 100.0 | 95.4 | 4.6 | 100.0 | 97.7 | 2.3 | 100.0 | 99.7 | 0.4 | 100.0 | 90.9 | 9.1 | 100.0 |
| 18.5-19.9 | N | 6816 | 152 | 6968 | 6560 | 408 | 6968 | 6755 | 199 | 6954 | 6846 | 108 | 6954 | 6659 | 295 | 6954 | 6797 | 157 | 6954 | 6941 | 27 | 6968 | 6434 | 520 | 6954 |
|  | % | 97.8 | 2.2 | 100.0 | 94.1 | 5.9 | 100.0 | 97.1 | 2.9 | 100.0 | 98.5 | 1.6 | 100.0 | 95.8 | 4.2 | 100.0 | 97.7 | 2.3 | 100.0 | 99.6 | 0.4 | 100.0 | 92.5 | 7.5 | 100.0 |
| 20.0-22.4 | N | 23079 | 547 | 23626 | 22206 | 1420 | 23626 | 22945 | 652 | 23597 | 23237 | 360 | 23597 | 22492 | 1105 | 23597 | 23038 | 559 | 23597 | 23529 | 97 | 23626 | 21814 | 1783 | 23597 |
|  | % | 97.7 | 2.3 | 100.0 | 94.0 | 6.0 | 100.0 | 97.2 | 2.8 | 100.0 | 98.5 | 1.5 | 100.0 | 95.3 | 4.7 | 100.0 | 97.6 | 2.4 | 100.0 | 99.6 | 0.4 | 100.0 | 92.4 | 7.6 | 100.0 |
| 22.5-24.9 | N | 20279 | 471 | 20750 | 19525 | 1225 | 20750 | 20223 | 502 | 20725 | 20402 | 323 | 20725 | 19754 | 971 | 20725 | 20238 | 487 | 20725 | 20628 | 121 | 20749 | 19098 | 1627 | 20725 |
|  | % | 97.7 | 2.3 | 100.0 | 94.1 | 5.9 | 100.0 | 97.6 | 2.4 | 100.0 | 98.4 | 1.6 | 100.0 | 95.3 | 4.7 | 100.0 | 97.7 | 2.4 | 100.0 | 99.4 | 0.6 | 100.0 | 92.2 | 7.9 | 100.0 |
| 25.0-27.4 | N | 9964 | 265 | 10229 | 9527 | 702 | 10229 | 9946 | 274 | 10220 | 10012 | 208 | 10220 | 9611 | 609 | 10220 | 9971 | 249 | 10220 | 10163 | 66 | 10229 | 9441 | 779 | 10220 |
|  | % | 97.4 | 2.6 | 100.0 | 93.1 | 6.9 | 100.0 | 97.3 | 2.7 | 100.0 | 98.0 | 2.0 | 100.0 | 94.0 | 6.0 | 100.0 | 97.6 | 2.4 | 100.0 | 99.4 | 0.7 | 100.0 | 92.4 | 7.6 | 100.0 |
| 27.5-29.9 | N | 3987 | 125 | 4112 | 3728 | 384 | 4112 | 3973 | 135 | 4108 | 3983 | 125 | 4108 | 3813 | 295 | 4108 | 3965 | 143 | 4108 | 4076 | 36 | 4112 | 3758 | 350 | 4108 |
|  | % | 97.0 | 3.0 | 100.0 | 90.7 | 9.3 | 100.0 | 96.7 | 3.3 | 100.0 | 97.0 | 3.0 | 100.0 | 92.8 | 7.2 | 100.0 | 96.5 | 3.5 | 100.0 | 99.1 | 0.9 | 100.0 | 91.5 | 8.5 | 100.0 |
| 30.0-34.9 | N | 2823 | 127 | 2950 | 2580 | 370 | 2950 | 2818 | 128 | 2946 | 2819 | 127 | 2946 | 2682 | 264 | 2946 | 2849 | 97 | 2946 | 2918 | 32 | 2950 | 2639 | 307 | 2946 |
|  | % | 95.7 | 4.3 | 100.0 | 87.5 | 12.5 | 100.0 | 95.7 | 4.3 | 100.0 | 95.7 | 4.3 | 100.0 | 91.0 | 9.0 | 100.0 | 96.7 | 3.3 | 100.0 | 98.9 | 1.1 | 100.0 | 89.6 | 10.4 | 100.0 |
| 35.0-39.9 | N | 680 | 38 | 718 | 599 | 119 | 718 | 679 | 39 | 718 | 675 | 43 | 718 | 638 | 80 | 718 | 699 | 19 | 718 | 705 | 13 | 718 | 632 | 86 | 718 |
|  | % | 94.7 | 5.3 | 100.0 | 83.4 | 16.6 | 100.0 | 94.6 | 5.4 | 100.0 | 94.0 | 6.0 | 100.0 | 88.9 | 11.1 | 100.0 | 97.4 | 2.7 | 100.0 | 98.2 | 1.8 | 100.0 | 88.0 | 12.0 | 100.0 |
| >=40.0 | N | 175 | 17 | 192 | 133 | 59 | 192 | 176 | 15 | 191 | 164 | 27 | 191 | 164 | 27 | 191 | 186 | 5 | 191 | 191 | 1 | 192 | 161 | 30 | 191 |
|  | % | 91.2 | 8.9 | 100.0 | 69.3 | 30.7 | 100.0 | 92.2 | 7.9 | 100.0 | 85.9 | 14.1 | 100.0 | 85.9 | 14.1 | 100.0 | 97.4 | 2.6 | 100.0 | 99.5 | 0.5 | 100.0 | 84.3 | 15.7 | 100.0 |
| Total | N | 69998 | 1800 | 71798 | 66946 | 4852 | 71798 | 69690 | 2017 | 71707 | 70358 | 1349 | 71707 | 67961 | 3746 | 71707 | 69938 | 1769 | 71707 | 71394 | 403 | 71797 | 66019 | 5688 | 71707 |
|  | % | 97.5 | 2.5 | 100.0 | 93.2 | 6.8 | 100.0 | 97.2 | 2.8 | 100.0 | 98.1 | 1.9 | 100.0 | 94.8 | 5.2 | 100.0 | 97.5 | 2.5 | 100.0 | 99.4 | 0.6 | 100.0 | 92.1 | 7.9 | 100.0 |
| Mean |  | 247.76 |  |  | 7.14 |  |  | 4.30 |  |  | 2.14 |  |  | 0.51 |  |  | 0.15 |  |  | 5.18 |  |  | 0.05 |  |  |
| SE (Mean) |  | 0.195 |  |  | 0.007 |  |  | 0.006 |  |  | 0.002 |  |  | 0.001 |  |  | 0.000 |  |  | 0.001 |  |  | 0.000 |  |  |
| Median |  | 244 |  |  | 6.9 |  |  | 4 |  |  | 2.08 |  |  | 0.48 |  |  | 0.12 |  |  | 5.17 |  |  | 0.04 |  |  |
| Max |  | 1980 |  |  | 23.1 |  |  | 19.09 |  |  | 14.39 |  |  | 3.26 |  |  | 4.75 |  |  | 8.3 |  |  | 0.54 |  |  |
| Min |  | 32 |  |  | 2.5 |  |  | 0.8 |  |  | 0.4 |  |  | 0.1 |  |  | 0 |  |  | 3.28 |  |  | 0 |  |  |
| SD |  | 52.21 |  |  | 1.77 |  |  | 1.55 |  |  | 0.57 |  |  | 0.16 |  |  | 0.13 |  |  | 0.33 |  |  | 0.03 |  |  |
| Skewness |  | 1.45 |  |  | 1.14 |  |  | 1.50 |  |  | 1.30 |  |  | 1.31 |  |  | 5.28 |  |  | 0.33 |  |  | 1.50 |  |  |
| Kurtosis |  | 31.30 |  |  | 5.87 |  |  | 7.31 |  |  | 14.33 |  |  | 7.76 |  |  | 91.58 |  |  | 4.13 |  |  | 9.51 |  |  |

Appendix Table 2: Blood parameters per BMI category. For each parameter the measures and percentages per BMI category are given, above and below the threshold

|  |  | CRP | | | TCL | | | Ferritin | | | Hemoglobin | | | Glucose Fasting | | | ALT | | | Creatinin |  |  |
| --- | --- | --- | --- | --- | --- | --- | --- | --- | --- | --- | --- | --- | --- | --- | --- | --- | --- | --- | --- | --- | --- | --- |
| BMI (kg/m2) |  | <5.0mg/l | >=5.0mg/l | Total | <5.17mmol | >=5.17mmol | Total | >=30µg/l | <30µg/l | Total | >=140g/l | <140g/l | Total | <5.5mmol/ | >=5.5mmol | Total | <56IU/L | >=56IU/L | Total | <=97umol/l | >97umol/l | Total |
| <17.0 | N | 253 | 10 | 263 | 255 | 8 | 263 | 248 | 15 | 263 | 247 | 14 | 261 | 184 | 29 | 213 | 261 | 2 | 263 | 256 | 7 | 263 |
|  | % | 96.2 | 3.8 | 100.0 | 97.0 | 3.0 | 100.0 | 94.3 | 5.7 | 100.0 | 94.6 | 5.4 | 100.0 | 86.4 | 13.6 | 100.0 | 99.2 | 0.8 | 100.0 | 97.3 | 2.7 | 100.0 |
| 17.0-18.4 | N | 1899 | 103 | 2002 | 1942 | 59 | 2001 | 1865 | 136 | 2001 | 1894 | 98 | 1992 | 1344 | 268 | 1612 | 1989 | 12 | 2001 | 1902 | 99 | 2001 |
|  | % | 94.9 | 5.1 | 100.0 | 97.1 | 3.0 | 100.0 | 93.2 | 6.8 | 100.0 | 95.1 | 4.9 | 100.0 | 83.4 | 16.6 | 100.0 | 99.4 | 0.6 | 100.0 | 95.1 | 5.0 | 100.0 |
| 18.5-19.9 | N | 6660 | 352 | 7012 | 6750 | 249 | 6999 | 6548 | 448 | 6996 | 6704 | 264 | 6968 | 4806 | 902 | 5708 | 6917 | 80 | 6997 | 6611 | 388 | 6999 |
|  | % | 95.0 | 5.0 | 100.0 | 96.4 | 3.6 | 100.0 | 93.6 | 6.4 | 100.0 | 96.2 | 3.8 | 100.0 | 84.2 | 15.8 | 100.0 | 98.9 | 1.1 | 100.0 | 94.5 | 5.5 | 100.0 |
| 20.0-22.4 | N | 22609 | 1141 | 23750 | 22535 | 1173 | 23708 | 22489 | 1216 | 23705 | 22961 | 665 | 23626 | 16272 | 2802 | 19074 | 23351 | 356 | 23707 | 21860 | 1848 | 23708 |
|  | % | 95.2 | 4.8 | 100.0 | 95.1 | 5.0 | 100.0 | 94.9 | 5.1 | 100.0 | 97.2 | 2.8 | 100.0 | 85.3 | 14.7 | 100.0 | 98.5 | 1.5 | 100.0 | 92.2 | 7.8 | 100.0 |
| 22.5-24.9 | N | 19781 | 1083 | 20864 | 19347 | 1489 | 20836 | 20032 | 800 | 20832 | 20248 | 502 | 20750 | 14190 | 2477 | 16667 | 20249 | 584 | 20833 | 18918 | 1918 | 20836 |
|  | % | 94.8 | 5.2 | 100.0 | 92.9 | 7.2 | 100.0 | 96.2 | 3.8 | 100.0 | 97.6 | 2.4 | 100.0 | 85.1 | 14.9 | 100.0 | 97.2 | 2.8 | 100.0 | 90.8 | 9.2 | 100.0 |
| 25.0-27.4 | N | 9617 | 653 | 10270 | 9057 | 1208 | 10265 | 9993 | 267 | 10260 | 10052 | 177 | 10229 | 6768 | 1374 | 8142 | 9523 | 741 | 10264 | 9246 | 1019 | 10265 |
|  | % | 93.6 | 6.4 | 100.0 | 88.2 | 11.8 | 100.0 | 97.4 | 2.6 | 100.0 | 98.3 | 1.7 | 100.0 | 83.1 | 16.9 | 100.0 | 92.8 | 7.2 | 100.0 | 90.1 | 9.9 | 100.0 |
| 27.5-29.9 | N | 3792 | 349 | 4141 | 3413 | 719 | 4132 | 4054 | 77 | 4131 | 4041 | 71 | 4112 | 2641 | 639 | 3280 | 3547 | 584 | 4131 | 3754 | 378 | 4132 |
|  | % | 91.6 | 8.4 | 100.0 | 82.6 | 17.4 | 100.0 | 98.1 | 1.9 | 100.0 | 98.3 | 1.7 | 100.0 | 80.5 | 19.5 | 100.0 | 85.9 | 14.1 | 100.0 | 90.9 | 9.2 | 100.0 |
| 30.0-34.9 | N | 2623 | 356 | 2979 | 2302 | 673 | 2975 | 2927 | 48 | 2975 | 2918 | 32 | 2950 | 1801 | 517 | 2318 | 2221 | 754 | 2975 | 2719 | 256 | 2975 |
|  | % | 88.1 | 12.0 | 100.0 | 77.4 | 22.6 | 100.0 | 98.4 | 1.6 | 100.0 | 98.9 | 1.1 | 100.0 | 77.7 | 22.3 | 100.0 | 74.7 | 25.3 | 100.0 | 91.4 | 8.6 | 100.0 |
| 35.0-39.9 | N | 559 | 173 | 732 | 523 | 207 | 730 | 723 | 7 | 730 | 708 | 10 | 718 | 403 | 152 | 555 | 444 | 286 | 730 | 689 | 41 | 730 |
|  | % | 76.4 | 23.6 | 100.0 | 71.6 | 28.4 | 100.0 | 99.0 | 1.0 | 100.0 | 98.6 | 1.4 | 100.0 | 72.6 | 27.4 | 100.0 | 60.8 | 39.2 | 100.0 | 94.4 | 5.6 | 100.0 |
| >=40.0 | N | 109 | 85 | 194 | 141 | 52 | 193 | 191 | 2 | 193 | 189 | 3 | 192 | 96 | 37 | 133 | 100 | 93 | 193 | 190 | 3 | 193 |
|  | % | 56.2 | 43.8 | 100.0 | 73.1 | 26.9 | 100.0 | 99.0 | 1.0 | 100.0 | 98.4 | 1.6 | 100.0 | 72.2 | 27.8 | 100.0 | 51.8 | 48.2 | 100.0 | 98.5 | 1.6 | 100.0 |
| Total | N | 67902 | 4305 | 72207 | 66265 | 5837 | 72102 | 69070 | 3016 | 72086 | 69962 | 1836 | 71798 | 48505 | 9197 | 57702 | 68602 | 3492 | 72094 | 66145 | 5957 | 72102 |
|  | % | 94.0 | 6.0 | 100.0 | 91.9 | 8.1 | 100.0 | 95.8 | 4.2 | 100.0 | 97.4 | 2.6 | 100.0 | 84.1 | 15.9 | 100.0 | 95.2 | 4.8 | 100.0 | 91.7 | 8.3 | 100.0 |
| Mean |  | 1.34 |  |  | 4.09 |  |  | 92.90 |  |  | 156.28 |  |  | 4.75 |  |  | 27.46 |  |  | 80.86 |  |  |
| SE(Mean) |  | 0.016 |  |  | 0.003 |  |  | 0.202 |  |  | 0.032 |  |  | 0.004 |  |  | 0.067 |  |  | 0.041 |  |  |
| Median |  | 0.2 |  |  | 4 |  |  | 82 |  |  | 156 |  |  | 4.7 |  |  | 23 |  |  | 80 |  |  |
| Max |  | 154 |  |  | 11.3 |  |  | 1269 |  |  | 217 |  |  | 35.1 |  |  | 1212 |  |  | 410 |  |  |
| Min |  | 0.1 |  |  | 1.1 |  |  | 1 |  |  | 67 |  |  | 1.5 |  |  | 1 |  |  | 41 |  |  |
| SD |  | 4.30 |  |  | 0.74 |  |  | 54.18 |  |  | 8.66 |  |  | 0.87 |  |  | 18.11 |  |  | 10.99 |  |  |
| Skewness |  | 10.26 |  |  | 0.72 |  |  | 2.44 |  |  | -0.09 |  |  | 2.23 |  |  | 9.39 |  |  | 0.96 |  |  |
| Kurtosis |  | 172.52 |  |  | 4.65 |  |  | 21.55 |  |  | 3.81 |  |  | 51.23 |  |  | 330.14 |  |  | 14.91 |  |  |
