# Supplementary material for: Increasing variability of body mass and health correlates in Swiss conscripts, a possible role of relaxed natural selection?
Source: Evol Med Public Health. 2018 Apr 28;2018(1):116–26. doi: 10.1093/emph/eoy012 (PMC6007356; doi:10.1093/emph/eoy012)
Supplement: Supplementary Data [file eoy012_supp.zip › Appendix Tables.pdf]

Appendix Table 1: Blood cell counts per BMI category. For each cell type the numbers and percentages per BMI category are given, above and below the threshold

		Thrombocytes			Leukocytes			Neutrophils			Lymphocytes			Monocytes			Eosinophils			Erythrocytes			Basophils		
BMI (kg/m2)		<360G /l	>=360 G /l	Total	<10G /l	>=10G /l	Total	<=8.0 G /l	>8.0G/ l	Total	<=3.5 G /l	>3.5G /l	Total	<0.8G /l	>=0.8 G /l	Total	<0.47 G /l	>=0.47 G /l	Total	0	>=6.1 T /l	Total	<8%	>=8%	Total
<17.0	N	257	4	261	237	24	261	249	11	260	254	6	260	252	8	260	252	8	260	258	3	261	235	25	260
	%	98.5	1.5	100.0	90.8	9.2	100.0	95.8	4.2	100.0	97.7	2.3	100.0	96.9	3.1	100.0	96.9	3.1	100.0	98.9	1.2	100.0	90.4	9.6	100.0
17.0-18.4	N	1938	54	1992	1851	141	1992	1926	62	1988	1966	22	1988	1896	92	1988	1943	45	1988	1985	7	1992	1807	181	1988
	%	97.3	2.7	100.0	92.9	7.1	100.0	96.9	3.1	100.0	98.9	1.1	100.0	95.4	4.6	100.0	97.7	2.3	100.0	99.7	0.4	100.0	90.9	9.1	100.0
18.5-19.9	N	6816	152	6968	6560	408	6968	6755	199	6954	6846	108	6954	6659	295	6954	6797	157	6954	6941	27	6968	6434	520	6954
	%	97.8	2.2	100.0	94.1	5.9	100.0	97.1	2.9	100.0	98.5	1.6	100.0	95.8	4.2	100.0	97.7	2.3	100.0	99.6	0.4	100.0	92.5	7.5	100.0
20.0-22.4	N	23079	547	23626	22206	1420	23626	22945	652	23597	23237	360	23597	22492	1105	23597	23038	559	23597	23529	97	23626	21814	1783	23597
	%	97.7	2.3	100.0	94.0	6.0	100.0	97.2	2.8	100.0	98.5	1.5	100.0	95.3	4.7	100.0	97.6	2.4	100.0	99.6	0.4	100.0	92.4	7.6	100.0
22.5-24.9	N	20279	471	20750	19525	1225	20750	20223	502	20725	20402	323	20725	19754	971	20725	20238	487	20725	20628	121	20749	19098	1627	20725
	%	97.7	2.3	100.0	94.1	5.9	100.0	97.6	2.4	100.0	98.4	1.6	100.0	95.3	4.7	100.0	97.7	2.4	100.0	99.4	0.6	100.0	92.2	7.9	100.0
25.0-27.4	N	9964	265	10229	9527	702	10229	9946	274	10220	10012	208	10220	9611	609	10220	9971	249	10220	10163	66	10229	9441	779	10220
	%	97.4	2.6	100.0	93.1	6.9	100.0	97.3	2.7	100.0	98.0	2.0	100.0	94.0	6.0	100.0	97.6	2.4	100.0	99.4	0.7	100.0	92.4	7.6	100.0
27.5-29.9	N	3987	125	4112	3728	384	4112	3973	135	4108	3983	125	4108	3813	295	4108	3965	143	4108	4076	36	4112	3758	350	4108
	%	97.0	3.0	100.0	90.7	9.3	100.0	96.7	3.3	100.0	97.0	3.0	100.0	92.8	7.2	100.0	96.5	3.5	100.0	99.1	0.9	100.0	91.5	8.5	100.0
30.0-34.9	N	2823	127	2950	2580	370	2950	2818	128	2946	2819	127	2946	2682	264	2946	2849	97	2946	2918	32	2950	2639	307	2946
	%	95.7	4.3	100.0	87.5	12.5	100.0	95.7	4.3	100.0	95.7	4.3	100.0	91.0	9.0	100.0	96.7	3.3	100.0	98.9	1.1	100.0	89.6	10.4	100.0
35.0-39.9	N	680	38	718	599	119	718	679	39	718	675	43	718	638	80	718	699	19	718	705	13	718	632	86	718
	%	94.7	5.3	100.0	83.4	16.6	100.0	94.6	5.4	100.0	94.0	6.0	100.0	88.9	11.1	100.0	97.4	2.7	100.0	98.2	1.8	100.0	88.0	12.0	100.0
>=40.0	N	175	17	192	133	59	192	176	15	191	164	27	191	164	27	191	186	5	191	191	1	192	161	30	191
	%	91.2	8.9	100.0	69.3	30.7	100.0	92.2	7.9	100.0	85.9	14.1	100.0	85.9	14.1	100.0	97.4	2.6	100.0	99.5	0.5	100.0	84.3	15.7	100.0
Total	N	69998	1800	71798	66946	4852	71798	69690	2017	71707	70358	1349	71707	67961	3746	71707	69938	1769	71707	71394	403	71797	66019	5688	71707
	%	97.5	2.5	100.0	93.2	6.8	100.0	97.2	2.8	100.0	98.1	1.9	100.0	94.8	5.2	100.0	97.5	2.5	100.0	99.4	0.6	100.0	92.1	7.9	100.0

Mean	247.76	7.14	4.30	2.14	0.51	0.15	5.18	0.05
SE (Mean)	0.195	0.007	0.006	0.002	0.001	0.000	0.001	0.000
Median	244	6.9	4	2.08	0.48	0.12	5.17	0.04
Max	1980	23.1	19.09	14.39	3.26	4.75	8.3	0.54
Min	32	2.5	0.8	0.4	0.1	0	3.28	0
SD	52.21	1.77	1.55	0.57	0.16	0.13	0.33	0.03
Skewness	1.45	1.14	1.50	1.30	1.31	5.28	0.33	1.50
Kurtosis	31.30	5.87	7.31	14.33	7.76	91.58	4.13	9.51

Appendix Table 2: Blood parameters per BMI category. For each parameter the measures and percentages per BMI category are given, above and below the threshold

		CRP			TCL			Ferritin			Hemoglobin			Glucose Fasting			ALT			Creatinin		
BMI (kg/m2)		<5.0mg/l	>=5.0mg/l	Total	<5.17mmol	>=5.17mmol	Total	>=30µg/l	<30µg/l	Total	>=140g/l	<140g/l	Total	<5.5mmol/	>=5.5mmol	Total	<56IU/L	>=56IU/L	Total	<=97umol/l	>97umol/l	Total
<17.0	N	253	10	263	255	8	263	248	15	263	247	14	261	184	29	213	261	2	263	256	7	263
	%	96.2	3.8	100.0	97.0	3.0	100.0	94.3	5.7	100.0	94.6	5.4	100.0	86.4	13.6	100.0	99.2	0.8	100.0	97.3	2.7	100.0
17.0-18.4	N	1899	103	2002	1942	59	2001	1865	136	2001	1894	98	1992	1344	268	1612	1989	12	2001	1902	99	2001
	%	94.9	5.1	100.0	97.1	3.0	100.0	93.2	6.8	100.0	95.1	4.9	100.0	83.4	16.6	100.0	99.4	0.6	100.0	95.1	5.0	100.0
18.5-19.9	N	6660	352	7012	6750	249	6999	6548	448	6996	6704	264	6968	4806	902	5708	6917	80	6997	6611	388	6999
	%	95.0	5.0	100.0	96.4	3.6	100.0	93.6	6.4	100.0	96.2	3.8	100.0	84.2	15.8	100.0	98.9	1.1	100.0	94.5	5.5	100.0
20.0-22.4	N	22609	1141	23750	22535	1173	23708	22489	1216	23705	22961	665	23626	16272	2802	19074	23351	356	23707	21860	1848	23708
	%	95.2	4.8	100.0	95.1	5.0	100.0	94.9	5.1	100.0	97.2	2.8	100.0	85.3	14.7	100.0	98.5	1.5	100.0	92.2	7.8	100.0
22.5-24.9	N	19781	1083	20864	19347	1489	20836	20032	800	20832	20248	502	20750	14190	2477	16667	20249	584	20833	18918	1918	20836
	%	94.8	5.2	100.0	92.9	7.2	100.0	96.2	3.8	100.0	97.6	2.4	100.0	85.1	14.9	100.0	97.2	2.8	100.0	90.8	9.2	100.0
25.0-27.4	N	9617	653	10270	9057	1208	10265	9993	267	10260	10052	177	10229	6768	1374	8142	9523	741	10264	9246	1019	10265
	%	93.6	6.4	100.0	88.2	11.8	100.0	97.4	2.6	100.0	98.3	1.7	100.0	83.1	16.9	100.0	92.8	7.2	100.0	90.1	9.9	100.0
27.5-29.9	N	3792	349	4141	3413	719	4132	4054	77	4131	4041	71	4112	2641	639	3280	3547	584	4131	3754	378	4132
	%	91.6	8.4	100.0	82.6	17.4	100.0	98.1	1.9	100.0	98.3	1.7	100.0	80.5	19.5	100.0	85.9	14.1	100.0	90.9	9.2	100.0
30.0-34.9	N	2623	356	2979	2302	673	2975	2927	48	2975	2918	32	2950	1801	517	2318	2221	754	2975	2719	256	2975
	%	88.1	12.0	100.0	77.4	22.6	100.0	98.4	1.6	100.0	98.9	1.1	100.0	77.7	22.3	100.0	74.7	25.3	100.0	91.4	8.6	100.0
35.0-39.9	N	559	173	732	523	207	730	723	7	730	708	10	718	403	152	555	444	286	730	689	41	730
	%	76.4	23.6	100.0	71.6	28.4	100.0	99.0	1.0	100.0	98.6	1.4	100.0	72.6	27.4	100.0	60.8	39.2	100.0	94.4	5.6	100.0
>=40.0	N	109	85	194	141	52	193	191	2	193	189	3	192	96	37	133	100	93	193	190	3	193
	%	56.2	43.8	100.0	73.1	26.9	100.0	99.0	1.0	100.0	98.4	1.6	100.0	72.2	27.8	100.0	51.8	48.2	100.0	98.5	1.6	100.0
Total	N	67902	4305	72207	66265	5837	72102	69070	3016	72086	69962	1836	71798	48505	9197	57702	68602	3492	72094	66145	5957	72102
	%	94.0	6.0	100.0	91.9	8.1	100.0	95.8	4.2	100.0	97.4	2.6	100.0	84.1	15.9	100.0	95.2	4.8	100.0	91.7	8.3	100.0
Mean		1.34			4.09			92.90			156.28			4.75			27.46			80.86		
SE(Mean)		0.016			0.003			0.202			0.032			0.004			0.067			0.041		

Median	0.2	4	82	156	4.7	23	80
Max	154	11.3	1269	217	35.1	1212	410
Min	0.1	1.1	1	67	1.5	1	41
SD	4.30	0.74	54.18	8.66	0.87	18.11	10.99
Skewness	10.26	0.72	2.44	-0.09	2.23	9.39	0.96
Kurtosis	172.52	4.65	21.55	3.81	51.23	330.14	14.91
