# Supplementary figures and images for: Increasing variability of body mass and health correlates in Swiss conscripts, a possible role of relaxed natural selection?
Source: Evol Med Public Health. 2018 Apr 28;2018(1):116–26. doi: 10.1093/emph/eoy012 (PMC6007356; doi:10.1093/emph/eoy012)

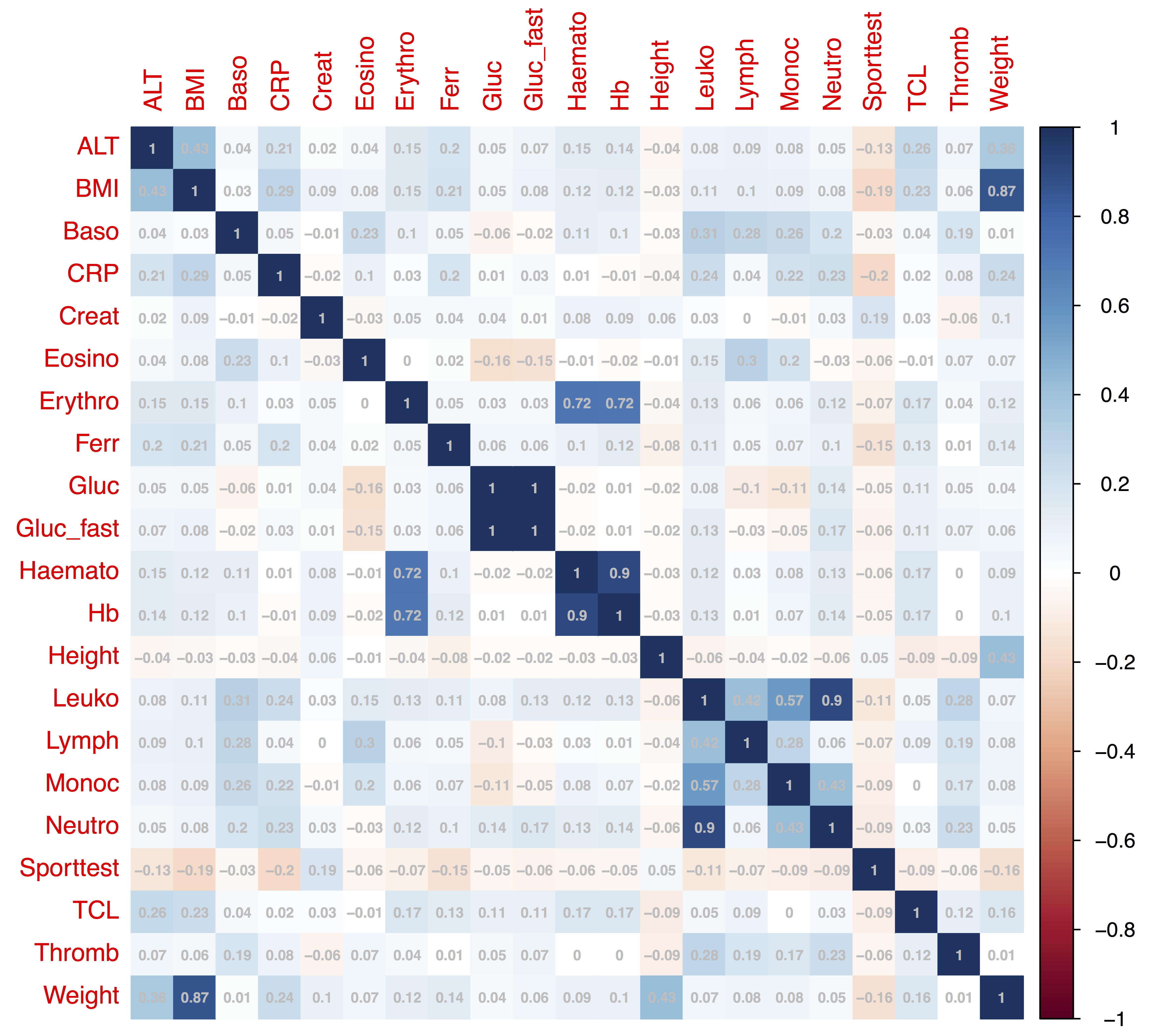

Supplement: Supplementary Data [file eoy012_supp.zip › AppendixFigure3.jpg]

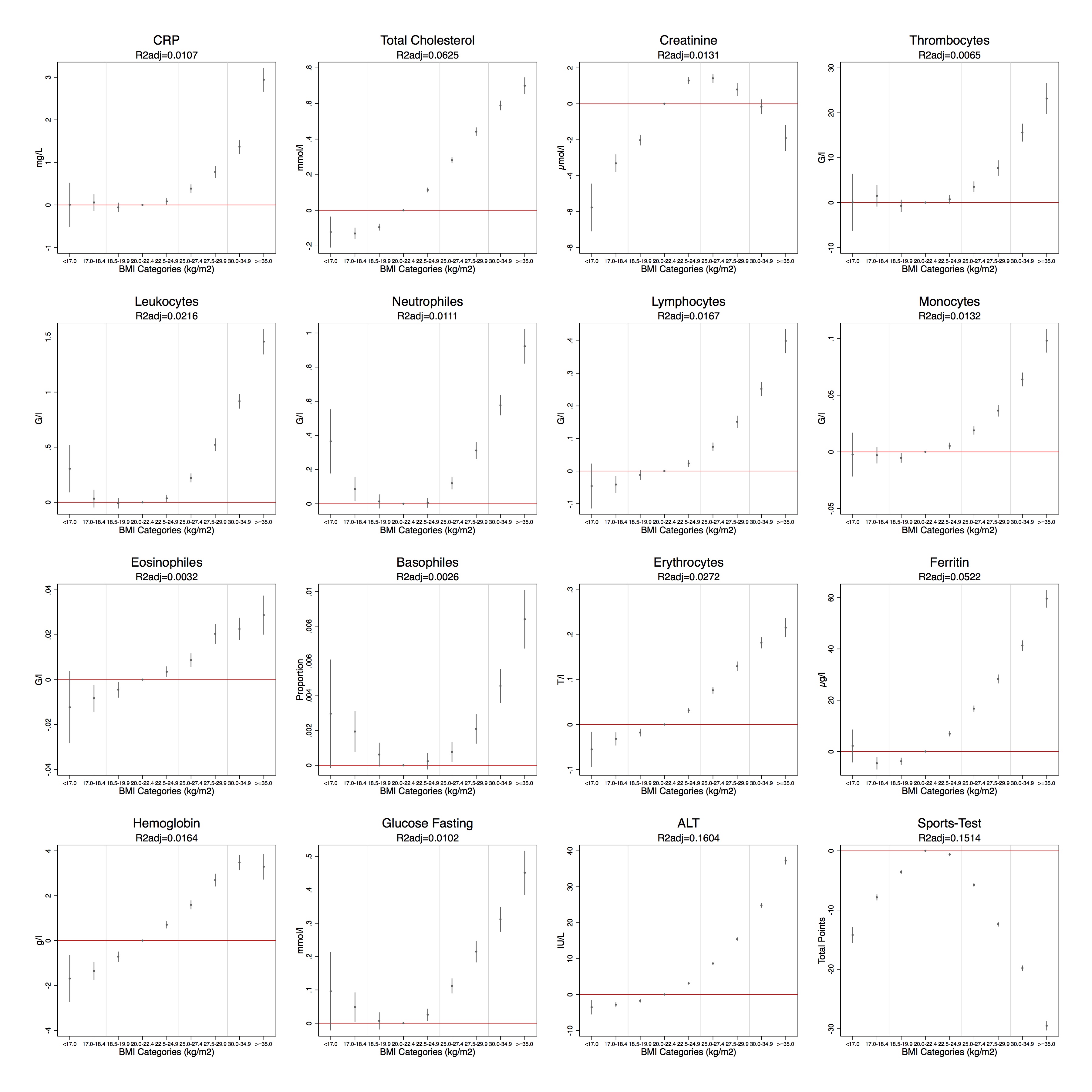

Supplement: Supplementary Data [file eoy012_supp.zip › AppendixFigure4.jpg]

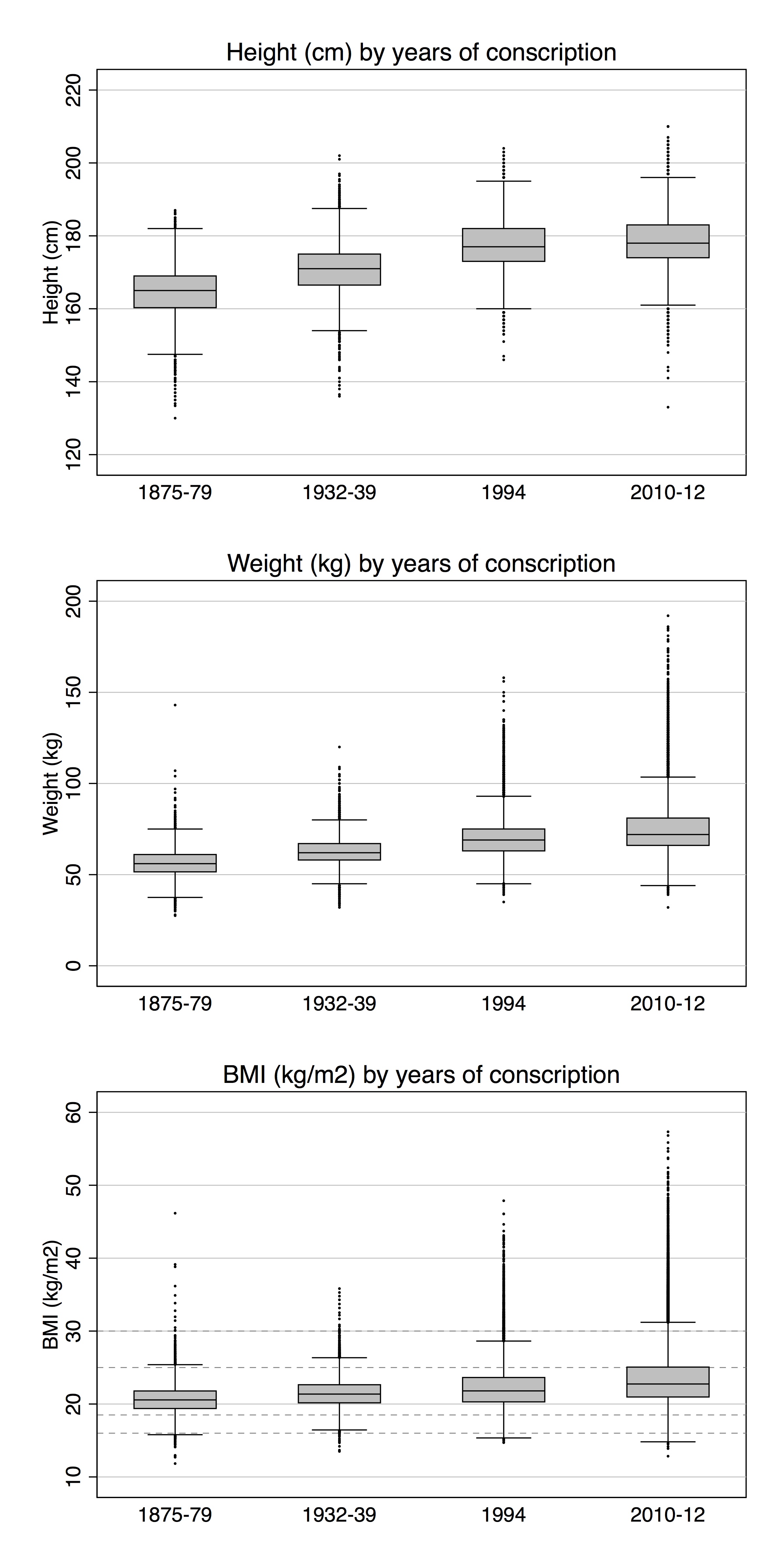

Supplement: Supplementary Data [file eoy012_supp.zip › AppendixFigure1.jpg]

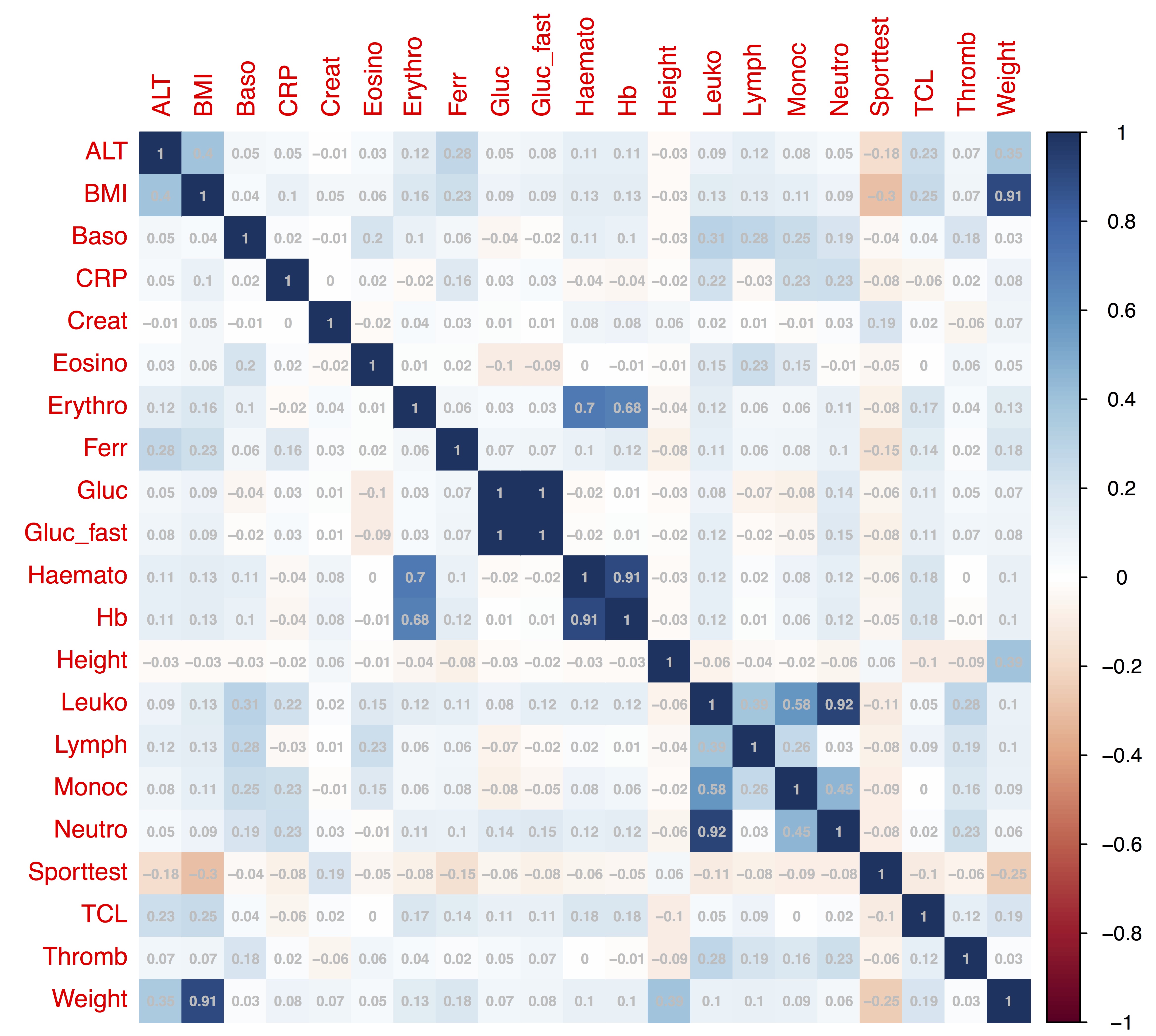

Supplement: Supplementary Data [file eoy012_supp.zip › AppendixFigure2.jpg]
